# The Relationship of Ketogenic Diet with Neurodegenerative and Psychiatric Diseases: A Scoping Review from Basic Research to Clinical Practice

**DOI:** 10.3390/nu15102270

**Published:** 2023-05-11

**Authors:** Maria Mentzelou, Antonios Dakanalis, Georgios K. Vasios, Maria Gialeli, Sousana K. Papadopoulou, Constantinos Giaginis

**Affiliations:** 1 Department of Food Science and Nutrition, School of Environment, University of Aegean, 81400 Myrina, Greece; fnsd22007@fns.aegean.gr (M.M.); vasios@aegean.gr (G.K.V.); gialeli.m@aegean.gr (M.G.); 2 School of Medicine and Surgery, University of Milano-Bicocca, 20900 Monza, Italy; 3 Department of Nutritional Sciences and Dietetics, School of Health Sciences, International Hellenic University, 57400 Thessaloniki, Greece; souzpapa@gmail.com

**Keywords:** ketogenic diet, neurodegenerative diseases, Alzheimer’s disease, Parkinson’s disease, cognitive impairment, depression, eating disorders, autism, nutritional intervention, ketosis

## Abstract

Background: The ketogenic diet (KD) has become widespread for the therapy of epileptic pathology in childhood and adulthood. In the last few decades, the current re-emergence of its popularity has focused on the treatment of obesity and diabetes mellitus. KD also exerts anti-inflammatory and neuroprotective properties, which could be utilized for the therapy of neurodegenerative and psychiatric disorders. Purpose: This is a thorough, scoping review that aims to summarize and scrutinize the currently available basic research performed in in vitro and in vivo settings, as well as the clinical evidence of the potential beneficial effects of KD against neurodegenerative and psychiatric diseases. This review was conducted to systematically map the research performed in this area as well as identify gaps in knowledge. Methods: We thoroughly explored the most accurate scientific web databases, e.g., PubMed, Scopus, Web of Science, and Google Scholar, to obtain the most recent in vitro and in vivo data from animal studies as well as clinical human surveys from the last twenty years, applying effective and characteristic keywords. Results: Basic research has revealed multiple molecular mechanisms through which KD can exert neuroprotective effects, such as neuroinflammation inhibition, decreased reactive oxygen species (ROS) production, decreased amyloid plaque deposition and microglial activation, protection in dopaminergic neurons, tau hyper-phosphorylation suppression, stimulating mitochondrial biogenesis, enhancing gut microbial diversity, restoration of histone acetylation, and neuron repair promotion. On the other hand, clinical evidence remains scarce. Most existing clinical studies are modest, frequently uncontrolled, and merely assess the short-term impacts of KD. Moreover, several clinical studies had large dropout rates and a considerable lack of compliance assessment, as well as an increased level of heterogeneity in the study design and methodology. Conclusions: KD can exert substantial neuroprotective effects via multiple molecular mechanisms in various neurodegenerative and psychiatric pathological states. Large, long-term, randomized, double-blind, controlled clinical trials with a prospective design are strongly recommended to delineate whether KD may attenuate or even treat neurodegenerative and psychiatric disease development, progression, and symptomatology.

## 1. Introduction

The classic ketogenic diet (KD) is defined as a diet with one gram of protein per kilogram of body weight, 10–15 g of carbohydrates daily, and the remaining calories from fat [1]. The purpose of this diet is to induce ketosis [2]. Several variations of the classical KD are presently in use. Currently, KD comprises typical KD, the medium chain triglyceride (MCT) diet, the modified Atkins diet (MAD), and low glycemic index treatment (LGIT) [1]. From a percentage point of view, in a KD, approximately 70–80% of energy is produced by fat, and the remaining 20–30% is covered by both proteins and carbohydrates [2]. During the KD, fatty acids are transformed to ketone bodies by hepatic metabolism and afterwards move into the blood circulation to promote nutritional ketosis, participating in several physiological or pathological responses [2]. Ketosis is considered to modify metabolic pathways to induce weight loss and potentially improve other health outcomes, such as a reduction in hyperglycemia and an improvement in lipid profiles [3]. Moreover, ketosis has been associated with the reduction of oxidation damage and the regulation of inflammation conditions. The products of ketone bodies’ metabolism are capable of covering about 80% of the brain’s energy requirements while additionally exerting neuroprotective properties [3]. As effective treatment of KD mainly depends on the patients’ compliance, collaboration amongst physicians, dieticians, the family environment, and patients is of great significance.

Low-carbohydrate diets have recently received attention from various international scientific organizations due to their feasibility and efficiency in treating mainly obesity and diabetes mellitus [4]. Low-carbohydrate diets such as KD can decrease insulin fluxes, causing an increased rate of lipolysis and resulting in increased fat breakdown [5]. Moreover, there has been significant attention paid to the use of KDs to treat type 2 diabetes in combination with obesity, improving hyperglycemia by decreasing circulating glucose and increasing insulin sensitivity [5]. KDs have also been shown to repeatedly decrease triglycerides and increase HDL-cholesterol levels, reducing cardiovascular disease risk [6,7]. Several randomized controlled clinical studies showed increased LDL-cholesterol during KD treatment in at least certain patient populations. However, in meta-analyses, the LDL-cholesterol response remained variable [4,6,7].

KD may also decrease cancer risk as it benefits from the decreased expression of ketolytic enzymes in tumor cells [8]. A substantial meta-analysis of 24 human studies indicated that KD could reduce tumor development and growth [9]. Accordingly, most of the animal studies supported the idea that KDs could exhibit anti-cancer properties. However, the currently available data concerning the anti-cancer impacts of KD in humans is still inadequate and restricted to specific cases. In this aspect, a probabilistic disagreement indicates that the existing data reinforces the concept of the anti-cancer impact assumption, at least for certain people [9].

KD can possibly enhance the genomic variety of the gut microbiota and raise the fraction of Bacteroidetes to Firmicutes [10]. Individuals affected by obesity represented an increase in Bacteroidetes, whereas Firmicutes were unaltered, and thus it seems that decreasing obesity through KD could lead to beneficial modifications in the gut microbiota [11]. Notably, recent research has proven a strong association between epileptic pathology and the gut microbiota [12]. In addition, the mechanisms that were involved in the well-known antiseizure actions of the KD in people with epilepsy could be affected by the gut microbiota [12].

Several surveys have noticed a substantially beneficial effect of KD intervention in the therapy of refractory epileptic pathology in both childhood and adulthood [13]. KD is currently a well-recognized therapeutic strategy for drug-resistant epilepsy with established efficiency. Notably, there is a gradually increasing number of studies supporting the idea that KD acts by affecting diminished adaptive and innate immunity, which arises in drug-tolerant epileptic pathology and in refractory status epilepticus [14]. Apart from epilepsy, there are still further possible applications in neuropsychiatric diseases, as KD seems to exhibit multiple anti-inflammatory, anti-oxidant, and neuroprotective activities [14]. Emergent findings support that KD may act effectively against neurodegenerative diseases like Alzheimer’s disease (AD), Parkinson’s disease (PD), amyotrophic lateral sclerosis (ALS), multiple sclerosis (MS), autism spectrum disorder (ASD), headache, and psychiatric diseases like depression, eating disorders, anxiety, bipolar disorder, and schizophrenia [15].

In this aspect, the current study intends to thoroughly summarize and scrutinize the currently available basic research performed in in vitro and in vivo settings, as well as the clinical evidence of the potential beneficial effects of classic KD against neurodegenerative and psychiatric diseases.

## 2. Methods

**Figure 1:**
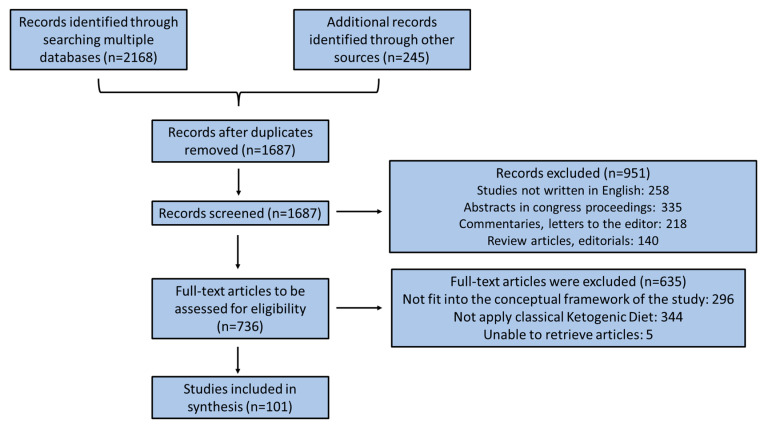
PRISMA Flow Diagram.

This is a scoping review that aims to map the literature on the relationship of KD with neurodegenerative and psychiatric diseases and provide an opportunity to identify key concepts, gaps in the research, and types and sources of evidence to inform practice, policymaking, and research on this topic. Peer-reviewed journal papers were included if they were: published between the periods of 2000 and 2023, written in English, involved in vitro and in vivo animal studies, as well as human participants, and described a measure for burden of treatment, e.g., including single measurements, measuring and/or incorporating one or two dimensions of burden of treatment. Quantitative, qualitative, and mixed-method studies were included to consider different aspects of measuring treatment burden. A comprehensive search of the existing international literature was carried out in the most accurate scientific databases, e.g., PubMed, Scopus, Web of Science, and Google Scholar, applying effective and characteristic keywords like ketogenic diet, ketosis, ketone bodies, neurodegenerative diseases, psychiatric diseases, Alzheimer’s disease, Parkinson’s disease, cognitive impairment, dementia, Huntington’s disease, autism spectrum disorder, eating disorders, anorexia and bulimia nervosa, binge eating, emotional eating, depression, schizophrenia, anxiety, stress, etc. The search was supplemented with the scanning of reference lists of relevant reviews and hand-searching key journals, commentaries, editorials, and abstracts in congress proceedings. The retrieved surveys were additionally comprehensively checked for related studies quoted in their text. The search strategies were drafted by an experienced librarian and further refined through team discussion by all authors. The final search results were exported into EndNote, and duplicates were removed by a library technician [Constantinos Gryparis]. All authors acted as reviewers. To increase consistency among reviewers, all reviewers screened all the retrieved publications, discussed the results, and amended the screening and data extraction manual before beginning screening for this review. Six reviewers working in pairs sequentially evaluated the titles, abstracts, and then full text of all publications identified by our searches for potentially relevant publications. We resolved disagreements on study selection and data extraction by consensus and discussion with all the authors/reviewers, if needed. A data charting form was jointly developed by two reviewers (G.K.V. and C.G.) to determine which variables to extract. The two reviewers independently charted the data, discussed the results, and continuously updated the data-charting form in an iterative process. Where we identified a systematic review, we counted the number of studies included in the review that potentially met our inclusion criteria and noted how many studies had been missed by our search. Inclusion criteria were any prospective, cross-sectional, descriptive, pilot, or case-report clinical studies conducted on Caucasian individuals. In vitro and in vivo animal models treated with KD were also included. Papers were excluded if they did not fit into the conceptual framework of the study. Only studies applying classical KD interventions that induce endogenous nutritional ketosis were included, whereas the MCT diet, MAD, and LGIT, as well as exogenous ketone supplementations, were excluded. The findings were selected based on relevance, and the most relevant ones were chosen and mentioned below according to the PRISMA flow diagram depicted in Figure 1. We examined emerging evidence on the potential beneficial effects of KD in a heterogenous group of diseases, such as neurodegenerative and psychiatric disorders, to help map the literature on this specific topic that may inform future research and systematic reviews on this issue.

## 3. Results and Discussion

### 3.1. Basic Research: In Vivo and In Vitro Studies Highlighting the Most Important Molecular Mechanisms of KD against Neurodegenerative and Psychiatric Diseases Development and Progression

**Figure 2:**
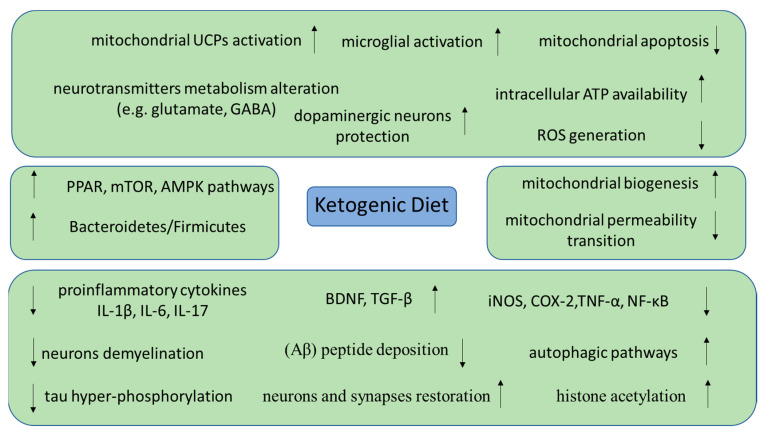
Molecular mechanisms through which KD can exert neuroprotective effects in vitro and in vivo (adenosine trisphosphate, ATP; reactive oxygen species, ROS; gamma-amino butyric acid, GABA; peroxisome proliferator activated receptor, PPAR; mammalian target of rapamycin, mTOR; 5′ adenosine monophosphate-activated protein, AMPK; interleukin, IL; brain-derived neurotrophic factor, BDNF; transforming growth factor beta, TGF-β; inducible nitric oxide synthase, iNOS; cycloogygenase-2, COX-2; tumor necrosis factor alpha, TNF-α; nuclear factor kappa B, NF-κB; uncoupling proteins, UCPs; increase, ↑; decrease, ↓).

The most important neuroprotective impacts of KD have been linked to multiple molecular mechanisms, as depicted in Figure 2. In an in vitro study, Beta-hydroxybutyrate acid (BHBA), the main ketone body generated throughout carbohydrates’ deprivation that arises in KD, was found to exert an essential impact on neuroprotection and prevention in neurodegenerative disorders, exerting promising therapeutic activity [16]. Microglial activation exerts a crucial impact on neurodegenerative disorders by inducing the release of several proinflammatory enzymes and cytokines [16]. In this aspect, BHBA affected BV2 microglial cells, stimulating microglial polarization to an M2 anti-inflammatory phenotype and decreasing migration’s capability after lipopolysaccharide (LPS) stimulation. Moreover, BHBA considerably lowered the production of the proinflammatory cytokine interleukin (IL)-17 and raised the levels of the anti-inflammatory cytokine IL-10 [16]. BHBA also substantially decreased LPS-stimulated protein and mRNA expression levels of inducible nitric oxide synthetase (iNOS), cyclooxygenase (COX)-2, tumor necrosis factor (TNF)-α, IL-1β, and IL-6 [17]. BHBA reduced LPS-induced degradation of inhibitor of NF-κB (IκB)-α and translocation of nuclear factor kappa B (NF-κB), whereas no effect was observed on mitogen-activated protein kinases (MAPKs) phosphorylation [17]. The above neuroprotective effects may occur by regulating the response of immune cells, such as by suppressing the activation of the nucleotide-binding domain and leucine-rich repeat (NLR)P3 inflammasome that is related to microglial inflammation progression [18]. NLRP3 inhibition was associated with reduced amounts of IL-1β and caspase-1, lessened reactive oxygen species (ROS) release, and lowered cellular mortality, as noted both in vitro and in vivo [19]. BHBA NF-κB inhibition led to a subsequent increase in glutathione synthesis [20], caused by increased nicotinamide adenine dinucleotide (NADH) oxidation [21].

In vivo, BHBA, functioning as an anti-inflammatory regulator, suppressed IL-6 and TNF-α production and induced brain-derived neurotrophic factor (BDNF) and transforming growth factor (TGF)-β generation in the brains of LPS-treated mice [22]. Thus, BHBA suppressed microglial progress retraction and depression-like behaviors, and these effects were eliminated by Akt suppression [22]. In vitro, BHBA suppressed IL-6 and TNF-α production, raised BDNF and TGF-β generation, decreased ROS levels, enhanced morphology alterations, and promoted the survival of LPS-stimulated BV2 cells [23]. In a transgenic mouse AD model, BHBA administration improved cognitive function by affecting multiple mechanisms, including being able to regulate dopamine neurons by inhibiting LPS-stimulated microglia triggering, both in vitro and in vivo, through facilitating the GPR109A signaling pathway [24]. BHBA diminished amyloid β (Aβ) peptide accumulation and microglial overactivation in the nervous system, enhancing mitochondrial respiratory function of hippocampus neurons and protecting them from Aβ toxicity [24]. In a rat PD model, KD provided protection in dopamine neurons against 6-hydroxydopamine (6-OHDA) neurotoxicity by increasing glutathione levels [25]. In a murine MS model, KD reduced disease development, enhanced motor disability and hippocampus atrophy, repaired lesions, and inhibited inflammation-related cytokines and ROS [26].

KD attenuated Aβ 40 and 42 accumulations in a mouse AD model after only 43 days of dietary intervention [27] and repaired motor deficits in transgenic ALS mice [28]. The later effects could be attributed to the capability of ketones to induce ATP production and skip suppression of complex I across the mitochondrial respiratory chain [28]. Similarly, 5 x familial Alzheimer’s disease (FAD) mice following a 16 week KD exhibited lower hippocampus Aβ accumulation than typical chow diets, which may be attributed to decreased microglia triggering and neuroinflammation [29]. Sixteen weeks of KD also enhanced spatial learning and memory as well as working memory in 5XFAD mice, a validated animal AD model [29]. Cognitive functions’ improvements were related to a repaired number of neurons and synapses in both the hippocampus and the cortex. In this animal model, KD intervention also lowered amyloid plaque accumulation and microglia triggering, leading to decreased neuroinflammation [29]. Kashiwaya et al. also showed that ketosis decreased both brain Aβ content and hyperphosphorylated tau in mouse AD models [30]. In addition, either inflammation or thermal nociception were considerably diminished by KD intervention in juvenile and adult rats [31]. In this aspect, KD exerts such neuroprotective roles by suppressing ROS release via stimulation of mitochondrial uncoupling proteins (UCPs) in juvenile mice [32].

There are various other mechanisms that may possibly cause the favorable impact of KD on AD. More to the point, aberrant glucose metabolism was noted even before the beginning of cognitive impairment in AD [33]. Positron emission tomography (PET) imaging analyses have documented that the use of glucose declined in AD brains, whereas ketone bodies’ usage did not [34]. Hence, KD may enhance cognitive function by supplying a supplementary fuel source to the nervous system. Moreover, apolipoprotein E (Apo-E) is considered an unfavorable AD hereditary risk factor, and the Apo-E ε4 allele was associated with decreased efficacy in fatty acid transfer and elevated AD prevalence [35]. So, KD may attenuate AD symptomatology by stimulating lipid metabolism.

Furthermore, several substantial pieces of evidence support the idea that KD could modify the gut microbiome [36]. The gut microbiome controls host brain functions through the gut-brain axis, which can exert crucial effects against AD pathogenesis [37]. The gut-brain axis links bidirectionally, and the gut microbiome can affect neurotransmission in the central nervous system (CNS) and vice versa. In a small clinical survey conducted on 25 MS patients, KD for 6 months positively affected the gut microbiome by improving gut microbial diversity [38]. Hence, KD may attenuate AD pathology by modulating the gut microbiome and lowering neuroinflammation. KD had considerable effects on modifying the gut microbiota to ameliorate disease symptomatology, mostly by increasing the Bacteroidetes to Firmicutes (B/F) fraction and decreasing proteobacteria in some cases [39]. Overall, clinical studies have supported the fact that the gut microbiota varied when KD intervention was applied; however, certain findings demonstrated that the microbial modifications were conflicting among different studies. This may be ascribed to the small sample size and the short period of the KD intervention.

KD for 16 weeks was also found to regulate the gut microbiota in young, healthy mice, and ketone bodies’ release was associated with the gut microbiota modifications [40]. KD significantly increased cerebral blood flow (CBF) and P-glycoprotein passages on the blood-brain barrier (BBB) to accelerate Aβ clearance [40]. These neurovascular enhancements were related to decreased mechanistic target of rapamycin (mTOR) and increased endothelial NOS (eNOS) protein expression levels. Moreover, KD raised the relative plethora of potential favorable gut microbiomes (*Akkermansia muciniphila* and *Lactobacillus*) and diminished that of potential pro-inflammatory taxa (Desulfovibrio and Turicibacter) [40].

Valproic acid (VPA) administration has been associated with elevated ASD prevalence in childhood, which is linked with problems in social behavior and connection and constrained monotonous interactions and interests. In vivo, animals treated with VPA showed enhanced social impairment, monotonous behavior, and an elevated nociceptive threshold [41]. Remarkably, animals co-treated with both VPA and KD presented improvements in social interaction. The above animals exhibited greater rates in the sociability index and social novelty index in comparison with the standard diet fed to VPA mice [41]. Another in vivo study determined whether KD may reverse the social impairments and mitochondrial dysfunction detected in a prenatal VPA rodent ASD model [42]. The offspring exposed to VPA prenatally showed a considerable reduction in the number of play initiations/attacks, which was inversed by KD intervention. Moreover, although prenatal VPA administration modified mitochondrial respiration, the KD was capable of restoring parts of bioenergetic disfunction. As KD is capable of modifying complex social behaviors and the mitochondrial respiratory system, it could constitute an effective therapeutic approach for ASD [42].

KD for 28 days suppressed mitochondria-facilitated apoptosis, probably by modulating acid-sensing ion channel 1a (ASIC1a), which facilitates Ca^2+^-dependent neuronal damage through acidosis, to exert neuroprotective effects and lead to excellent cognitive improvement in a rat model of temporal lobe epileptic pathology [43]. In KD-fed pentylenetetrazol (PTZ)-kindled rats, mitochondrial cytochrome c was elevated, cytosolic cytochrome c was diminished, and downstream cleaved caspase-3 was diminished, indicating a decrease in mitochondrial apoptosis [44]. Moreover, KD activated autophagy pathways and reduced brain damage during PTZ-kindled seizures. The neuroprotective KD impact seems to be mediated through a decrease in mitochondrial cytochrome c production [44].

In vivo, after 21 days of sleep deprivation, the wild-type one year old C57BL/6 female mice showed AD symptoms, followed by cognition impairment, Aβ accumulation, and tau hyperphosphorylation in the hippocampus. However, the above effects are able to be reversed by KD intervention, implying a protective KD impact on sleep deprivation (SD)-caused AD [45]. This preventive action of KD was associated with ferroptosis suppression and neuronal repair stimulation through the Sirt1/Nrf2 pathway in SD wild-type, one year old C57BL/6 female mice [45]. KD also ameliorated prolonged SD-stimulated cognitive impairment by suppressing hippocampus neuronal ferroptosis in young mice (7 weeks old) [46].

In the LPS-stimulated rat PD model, KD exerted an anti-inflammatory effect that was associated with the regulation of the Akt/GSK-3β/CREB signaling pathway facilitated by the histone acetylation of the mGluR5 promotor region [47]. Thus, targeting mGluR5 with epigenetic modification could be a promising approach to attenuate the microglia triggering in PD. Moreover, the KD needs to be initiated before the PD begins in high-risk populations to obtain an additional beneficial effect [47]. A survey assessed KD impacts in a rat PD model [48]. It was found that restricting glucose supply improved neuronal tolerance in the substantia nigra to damage and inhibited the development of PD symptomatology [48].

Ruskin et al. applied a KD in a transgenic mouse model of HD (R6/2 1J), with emphasis on its life-long behavior and physiological properties [49]. This study did not find any harmful effects of KD on any behavior characteristic examined (locomotor action and coordination, working recall capacity) or a considerable alteration in life expectancy. Although gradual weight decline is a conventional characteristic of HD, this study supported the hypothesis that the KD, which usually leads to weight decrease in control animals, slowed down the decrease in body weight of the transgenic mice [49].

An in vivo study investigated the impacts of KD intervention and routine voluntary exercise on anxiety and depressive behavior in Balb/c mice [50]. Both anxiety and depressive symptoms were reduced in KD-exercised mice. This decrease in anxiety and depressive behavior caused by KD and routine voluntary exercise could be related to enhanced BHBA amounts, reduced LDL/HDL fraction, and insulin or glucose concentrations [50].

In a Shank3 mouse model of autism, KD was also considered a favorable therapeutic approach for social deficits through restoring histone acetylation and gene expression in the nervous system [51]. A 4 week KD elevated histone acetylation levels in prefrontal cortex (PFC) neurons. Moreover, behavior analyses indicated that KD intervention extended the rescue of social preference impairments in Shank3-deficient mice [51].

### 3.2. Clinical Studies Evaluating the Potential Beneficial Impacts of KD Intervention in the Treatment and Management of Neurodegenerative and Psychiatric Diseases

#### 3.2.1. Alzheimer’s Disease

**Figure 3:**
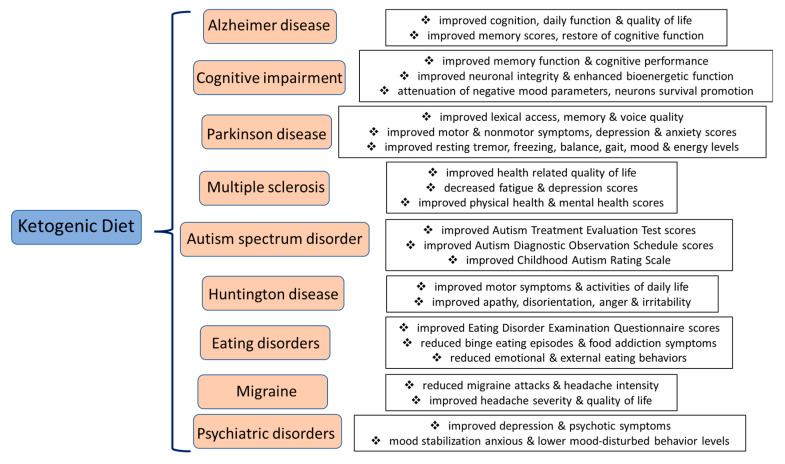
Potential beneficial impacts of KD intervention in the treatment and management of neurodegenerative and psychiatric diseases.

KD intervention may exert multiple favorable effects in the treatment and management of neurodegenerative and psychiatric diseases, as depicted in Figure 3. Precision medicine techniques have been applied using personalized, targeted, and multifactorial approaches for reversing cognitive decline [52]. As far as KD is concerned, in a randomized crossover trial, a 3 month modified KD in 21 individuals with AD significantly increased their AD Cooperative Study—Activities of Daily Living (ADCS—ADL) inventory and Quality of Life in AD (QOL-AD) questionnaire scores [53]. Participants with AD following KD exhibited merely a borderline correlation with enhanced cognitive function, which increased by about two points on the Addenbrookes Cognitive Examination-III (ACE-III) scale [53]. Thus, KD improved everyday performance status and quality of life, two factors of high significance for individuals diagnosed with dementia [53]. However, this clinical study had certain limitations. More to the point, the number of enrolled participants is small, while a short-term KD intervention was applied. Moreover, non-AD participants were not enrolled, while individuals with AD in the KD intervention group showed a moderate degree of weight loss compared to those following a conventional diet, and this could affect additional clinical outcomes [53].

A small clinical survey evaluated the effect of a 3 month KD intervention on 15 older adults with AD [54]. Amongst the 10 final completers, the mean of the AD Assessment Scale-cognitive subscale (ASCS) rating increased by about four points for the duration of KD intervention. Then, MCT-complemented KD was applied for a 1 month washout and returned to baseline next to the washout [54]. However, this study did not include any control groups, and therefore potential confounders might not be excluded [54]. A previous randomized controlled clinical survey (phase I/II) documented preliminary evidence on the effect of a 12 week MAD compared to a conventional nutritional intervention in 14 older adults diagnosed with mild cognitive impairment (MCI) or early AD [55]. Participants with high MAD compliance increased their memory scores; however, total diet compliance was just fair, and cognitive function did not improve [55].

In another small clinical survey, 17 older individuals (eleven with MCI and six with normal cognition status) were randomly assigned to adopt a modified KD for 6 weeks following a 6 week washout period [56]. This study identified specific gut microbiota signs that were linked with MCI, revealing that these signs correlated with certain disease biomarkers such as the accumulation of Aβ-40 and Aβ-42 and tau (total and phosphorylated) in the cerebrospinal fluid (CSF) of study participants [56]. Although this is a single-center, double-blinded study, it can support causality due to its cross-over design. A case study focused on a middle-aged woman with Down syndrome diagnosed with AD and the absence of seizures with progressive cognitive impairment over 6 years [57]. A KD reversed her cognition performance over 6 weeks, with a rise in her Activities of Daily Living Scale (ADLS) score [57].

#### 3.2.2. Cognitive Impairment

A small pilot study on 23 older individuals with MCI indicated that a modified KD for 6 weeks improved memory function in those with a high risk of AD [58]. In a following uncontrolled survey on five older adults with MCI, proton magnetic resonance spectroscopy (MRS) image analysis revealed considerably elevated myo-inositol and borderline rises of N-acetyl-aspartate and creatine plus phosphocreatine, along with enhanced cognition performance status [59]. This evidence supported the hypothesis that the KD was associated with enhanced autophagy, greater neuronal integrity, and increased bioenergetic function [59]. A phase I/II randomized clinical survey assessed the efficiency of applying a MAD intervention to stimulate ketogenesis in 27 individuals with MCI or early AD [55,60]. Among the participants of the MAD intervention group who showed at least low levels of urinary ketones, a considerably strong rise in the Memory Composite Score (MCS) between baseline and the 6 week evaluation was recorded [55,60]. A modified Mediterranean-KD regimen decreased amyloid beta, p181-tau, and neurofilament light in a randomized cross-over study of persons with MCI, and it specifically addressed the activation of different glutamate receptors [61].

In a 6 week controlled-KD intervention, no evidence of a constant decrease in mood or cognition performance was found in adults affected by overweight or obesity (*n* = 13) aged between 21 and 65 years [62]. The insertion of a ketone salt into a well-formulated hypocaloric KD attenuated negative mood parameters during the early intervention stages with no evidential variations in cognitive function [62]. A significant advantage of this survey was the presence of a controlled-nourishing design, which guarantees that participating individuals efficiently adopt the applied nutritional intervention [62]. On the other hand, this survey was performed on a military sample group, and its findings cannot be extrapolated to the general population [62]. In a small clinical survey of 20 older adults with subjective memory complaints (*n* = 11) or MCI (*n* = 9), two diets were followed, including a modified KD [63]. The modified KD resulted in improved CSF AD biomarkers’ profiles, as demonstrated by enhanced CSF Aβ42 (independently by grouping), reduced CSF tau (only in MCI), and elevated CSF Aβ42/tau fraction after modified KD (higher in MCI) [63]. This study had some limitations despite promising results. In fact, the rather small number of participants limited generalizability, reducing the ability to take into account subclass response factors like APOE genotype or gender.

Brain-derived neurotropic factor (BDNF) constitutes a crucial component of brain plasticity, which improves neuronal survival through growth and maturation, and BDNF expression is reduced in disease states related to cognitive decline and metabolic dysregulation [64]. In humans, fasting plasma BDNF levels decreased in individuals following KD compared to those that did not. Moreover, intense cycling exercise promptly increased plasma BDNF levels independently of ketosis. Body weight loss decreased fasting plasma BDNF levels independently of dietary components or ketosis levels [64]. The above findings highlighted the plasticity of plasma BDNF levels stimulated by lifestyle factors; however, they do not suggest any considerable relationship with temporally paired BHBA levels [64].

An exploratory, randomized, cross-over clinical trial was performed on seven male military personnel. Individuals followed an iso-energy KD or a carbohydrate-based nutritional intervention for two weeks. Then, 36 h of continued sleeplessness following a 12 day washout were applied [65]. BHBA was higher and glucose was lower in the KD group in comparison with the carbohydrate-based nutritional intervention group. KD enhanced psychomotor watchfulness task function, the running recall constant performance test, and vigor, fatigue, and sleepiness in comparison with the carbohydrate-based diet [65].

#### 3.2.3. Parkinson’s Disease

A small randomized-controlled clinical trial enrolled patients with MCI associated with PD in a 2 month dietary intervention with enrollment in either a high-carbohydrate intake characteristic of the Western diet model (*n* = 7) or a low-carbohydrate intake, KD (*n* = 7) [66]. The KD participants’ group showed improved vocabulary access and recall as well as a tendency for decreased interference in recall in comparison with the high-carbohydrate group [66]. Another pilot randomized controlled clinical trial was conducted on 47 individuals with PD who followed either a low-fat or a KD for 2 months [67]. This study demonstrated that both diets substantially enhanced motor and nonmotor symptomatology, but the KD group was characterized by higher improvements in nonmotor symptoms than the low-fat diet group [67].

In a small feasibility study, 5 of 7 participants with PD made a household “hyperketogenic” diet and adhered to it for about one month [68]. All five participants showed increased Unified Parkinson’s Disease Rating Scale (UPDRS) rates for the duration of hyperketonemia, as did symptoms like resting tremor, freezing, balance, gait, mood, and energy levels; however, a placebo effect was not taken into consideration [68]. These findings should be considered with attention, as this study had certain limitations, such as a small sample size and subjective ratings, while a control group to eliminate the placebo effect was absent. A comparative clinical study was conducted on 74 individuals with PD who reported a voice disorder associated with their disease and who followed either KD or a regular diet group for 3 months [69]. All mean Voice Handicap Index (VHI) characteristics improved in the KD group. Thus, KD could be considered an alternate therapeutic strategy to enhance the voice quality of people with PD [69].

A recent case report on a 68 year old woman with PD stage I and previous moderate anxiety and depressive symptomatology who followed a conventional KD for 24 weeks was performed [70]. Anxiety symptomatology at 12 and 24 weeks was improved, and small improvements on depressive scale scores at 24 weeks were noted [70]. A small pilot clinical study conducted on 16 adults ages 36–80 with PD also showed that a low carbohydrate and healthy fat KD intervention for 12 weeks resulted in significant improvements in Parkinson’s Anxiety Scale (PAS) rates and Part I of the UPDRS [71].

#### 3.2.4. Multiple Sclerosis

A small preliminary clinical study was performed to evaluate the safety and practicability of fasting mimicking diet (FMD) or KD on health-related quality of life (HRQOL) in randomly assigned individuals with relapsing-remitting MS [72]. In fact, 60 individuals with relapsing-remitting MS were enrolled at random in a conventional diet or a KD for a half year or a single cycle of modified FMD for one week that was then adopted as a Mediterranean dietary pattern for a half year. The KD cohort presented clinically significant increases in HRQOL rates at 12 weeks that comprised the whole quality of life modification in health, including the physical health component and the psychological health component [72]. KD in people with MS was safe, well tolerated, and resulted in high compliance [72]. However, MRI analyses, adequately blinded clinical assessments, and immune function analyses could greatly enhance the strength of these clinical findings.

In this context, the following randomized controlled clinical trial was conducted on 60 people with relapsing-remitting MS who were assigned to one of the below groups (*n* = 20 for each group): (a) adapted KD, (b) caloric restriction diet, and (c) control diet [73]. Six months of KD significantly reduced the production of the pro-inflammatory arachidonate 5-lipoxygenase (ALOX5), a crucial factor in pro-inflammatory leukotriene biosynthesis, and decreased the production of pro-inflammatory COX1 and COX2 [73]. KD in individuals with relapsing-remitting MS improved the Multiple Sclerosis Quality of Life (MSQOL) 54 index and lowered the peripheral lymphocyte count [73]. It should be noted that in individuals with MS, the ALOX5 path is associated with microglial triggering and neuroinflammation, contributing to axonal impairments and motor neuron dysregulation [74].

A small, open-label, single-arm clinical trial that assessed the safety and tolerability of KD revealed reduced BMI, body fat mass, fatigue, and depressive symptoms in 20 individuals with relapsing-remitting MS [75]. Nevertheless, the above study did not include a control group and had only a six month dietary intervention duration that does not establish reliable impacts on disease development [75]. Moreover, the above study exclusively enrolled people with relapsing, clinically, and radiographically constant MS, which prohibited any extrapolation to advanced MS subcategories or to individuals with actively relapsing MS, highlighting the need for future longitudinal studies with a randomized and case-control design to interpret the impact of KD on disease management [75]. More recently, in a two-phase clinical study, 65 individuals with relapsing MS were assigned to a half year longitudinal, intention-to-treat KD intervention [76]. MS patients showed substantial declines in fatty mass and an approximately 50% decrease in self-reported fatigue and depressive symptoms. MS QoL physical and mental health composite scores increased on diet. Substantial improvements in Expanded Disability Status Scale rates, the 6 min walk, and the Nine-Hole Peg Test were recorded [76]. Serum leptin decreased and adiponectin increased in the KD-treated group, revealing an adipose-associated inflammatory state in participants with relapsing MS [76]. The main disadvantage of the above survey is the absence of a paired control group that adopted a conventional dietary pattern. However, while this study lacked controls, its findings seem crucial for the upcoming phase III clinical study design [76]. In this aspect, in 24 healthy young people, following a very low-carbohydrate, high-fat diet combined with regular exercise for a 12 week duration led to a beneficial alteration in body weight status and fatty mass, along with favorable alterations in serum adiponectin and leptin concentrations [77].

In an ongoing single-center, controlled clinical trial, 111 individuals with relapsing-remitting MS following constant immunoregulatory treatment or no disease-regulating treatment are randomly assigned to one of three 18 month nutritional interventions, to which a KD with a limited daily carbohydrate consumption of 20–40 g is applied [78]. This clinical trial aims to evaluate the impact of a KD on MS development and progression [78]. This study has several strengths, including an adequate sample size and a randomized design blinded to any outcome assessment with a long duration of 18 months. Additionally, this study focuses on both MRI-measured disease activity and progression measures and various patient-associated endpoints like fatigue, depressive symptoms, and quality of life. The findings of this survey remain to be published [78].

#### 3.2.5. Autism Spectrum Disorder

A small longitudinal clinical survey on the effect of KD was performed on 30 children with autism aged from 4 to 10 years [79]. KD was utilized for a half year, with constant administration for one month, disrupted by two week diet-free periods. Among 18 of 30 children (60%) who remained on the diet, improvements were noted in various parameters in agreement with the Childhood Autism Rating Scale (CARS) [79]. Considerable improvements were noted in two patients, average improvements in eight patients, and minor improvements (2–8 units) in eight MS patients [79].

In an open-label, observer-blinded clinical trial, a modified gluten-free KD in conjunction with MCTs was applied to 15 children aged between 2 and 17 years with autism spectrum disorder (ASD) [80]. At 3 months, considerable improvement, characterized by a reduction of more than seven points in the Autism Diagnostic Observation Schedule, 2nd edition (ADOS-2) overall score, was noted in six children. Modest improvement was found (>3 points) in two children, and slight or no improvement (≤3 points) was detected in seven children [80]. Overall, 8 of 15 children who adhered to KD enhanced their total rate by at least four units. An increase in CARS rates was also documented at 3 months [80].

In a case-control study, 45 children with ASD at the age of 3–8 years old were distributed into three groups: one group followed modified Atkins KD, another consumed a gluten-free, casein-free (GFCF) diet, and the last group followed a conventional diet as a control [81]. At 6 months, the first two groups exhibited substantially improved Autism Treatment Evaluation Test (ATEC) and CARS rates than the control group. Notably, the KD group exhibited higher scores in cognition and sociability in comparison to the GFCG diet group [81].

Herbert and colleagues examined a 4 year old girl presenting serious progressive autism and previous epileptic pathology for whom medication treatment was not effective [82]. Following a GFCG KD, she exhibited a decrease in ASD symptomatology. During a follow-up of over 8 years, she obtained a reduction in her CARS rating from 49 to 17, demonstrating a shift from serious autism to no autism. She also showed a rise in her intelligence quotient (IQ) of about 70 units [82]. Another case report has been published concerning a 6 year old child presenting high-functioning autism and subclinical epilepsy discharges who did not respond adequately to various behavioral and psychopharmacological medications [83]. After one month following KD, the child’s performance and mental capacity were enhanced with respect to hyperactivity, attention period, no normal responses to visual and acoustic stimuli, objects’ usage, changes’ adaptation, communication capacity, fear, anxiety, and emotional responses. The above improved characteristics remained up to the end of the follow-up interval at 16 months on the KD [83].

#### 3.2.6. Huntington’s Disease

Huntington’s disease (HD) is a progressive, severe neurodegenerative disorder with inadequate therapy approaches. Several studies support the idea that mitochondrial disfunction in the nervous system and skeletal muscle is implicated in HD pathological processes. In this aspect, a case report study was performed on a 41 year old man with developing and worsening HD who followed a period-limited KD for 48 weeks [84]. He exhibited a 52% improvement in his motor symptoms, a 28% improvement with respect to his daily living activities, and a 20% improvement in his combined Unified HD Rating Scale (cUHDRS). Moreover, his HD-associated behavioral difficulties such as apathy, disorientation, anger, and irritability were improved by 50–100%, and his mood-associated quality of life by 25% [84].

#### 3.2.7. Eating Disorders

##### Anorexia Nervosa

In a small pilot case study, five individuals who regained weight after anorexia nervosa but with chronic constant eating disease psychopathology implemented a therapeutic KD to maintain body weight [85]. After experiencing nutritional ketosis, individuals were treated with six ketamine infusions and were then followed for around 6 months. Two individuals adopted KD for 2 months before ketamine treatment because of a great behavioral response and continued KD, while three individuals followed KD for one month before and during ketamine and then diminished gradually on the last infusion [85]. The study group exhibited substantial advancements on the Clinical Impairment Assessment, Eating Disorder Examination Questionnaire (EDEQ) Global Rating, EDEQ-Eating Concerns, EDEQ-Weight Concerns, EDEQ-Shape Concerns, Eating Disorders Recovery Questionnaire (EDRQ) Acceptance of Self and Body, and EDRQ-Social and Emotional Connection [85]. Scolnick and colleagues [86] performed a case study on the impacts of KD on a 29 year old woman with a 15 year history of anorexia nervosa. A KD of 2:1 to 1:1 relative rate of fats to carbohydrates was applied, and a KD dietitian supported nutritional training. Following 3 months of KD, the patient showed some improvement; however, she was still suffering from anorexia-related symptoms [86].

##### Binge Eating

In a case report, three patients affected by obesity (age 34, 54, and 63 years; mean BMI 43.5 kg/m^2^) and suffering from comorbid binge eating and foodstuffs’ dependence symptomatology followed a KD for a period of 6–7 months [87]. Participants documented considerable decreases in binge eating incidents and foodstuffs’ dependence symptoms concerning cravings and absence of control as determined by the Binge-Eating Scale (BES), Yale Food Addiction Scale (YFAS), or Yale-Brown Obsessive-Compulsive Scale (Y-OB-CS) modified for binge eating, depending on the case [87]. In a small pilot study, five female individuals suffering from binge eating and/or foodstuff dependency symptomatology followed a very low-calorie KD with protein alternatives for a duration of 5–7 weeks and later followed a low-calorie diet for 11–21 weeks [88]. All participating women showed reduced food dependence and/or BES rates [88].

##### Emotional Eating

Decreases in emotional and external eating performances in 35 adult individuals affected by obesity were associated with increased BHBA levels and a reduction in peripheral neuropeptide Y (pNPY) following 12 weeks of KD [89]. Moreover, BHBA is negatively associated with pNPY levels. The above relationships did not depend on obesity markers like fatty mass, reduced carbohydrate consumption, or elevated fat consumption, or other stress-associated indicators like cortisol, which supports a potential effect of BHBA in modulating the detected emotional and functional responses [89]. The same research group found that 12 week KD resulted in decreased appetite, reduced emotional and external eating, enhanced body-look satisfaction, and favorable physical functioning in 35 obese adults [90]. A significant increase in BDNF levels was noted in the first 2 weeks, which then went back to baseline. Elevated NPY levels were also noted throughout the duration of the 12 week KD [90].

#### 3.2.8. Migraine

Migraine is considered a common comorbidity in several neurodegenerative diseases and is characterized by similar pathological disease mechanisms, including inflammation and microglial overexpression [91,92]. There are currently several pieces of evidence demonstrating an association between migraine and an increased risk of cognitive impairment [93,94,95]. Refractory migraine constitutes a mainly incapacitating type of prolonged migraine that does not respond to various preventive approaches. In a small clinical trial, 22 individuals with refractory migraine were enrolled. Thirteen patients were recruited in the KD arm, and eight were not eligible for KD and went on a low-carb diet [96]. Patients following KD exhibited a substantial decrease in the incidence of migraine attacks, headache strength, and medication dose, whereas no considerable decrease was noted in the low-carb diet group. An association between ketone generation and the impact of headaches was found among individuals who more frequently adopted KD [96]. Moreover, a 3 month KD intervention on 50 individuals led to a decline in the painful symptoms of medication-refractory prolonged migraine [97]. In fact, the number of days with symptoms reduced from 30 (the median value) to 7.5, and the period of the migraine incidents lowered from 24 h (the median value) to 5.5 h. The participants’ pain level, first at maximum value for 83% of the patients, became better for 55% of them, and medications taken in 4 weeks were reduced from thirty (median value) to six doses [97].

In a randomized, double-blinded, cross-over clinical trial, a one month period of very low-calorie KD, despite resulting in comparable weight decline and glycemic profile, was considerably more efficient compared to a very low-calorie diet without KD in reducing the risk of migraine attacks [98]. The above was proved by a reduction in the prevalence of migraine days and attacks in 35 people with overweight or obesity and migraine [98]. Moreover, a 3 month randomized controlled crossover clinical survey including two nutritional intervention phases was undertaken in 16 individuals [99]. Eligible participants had previous migraines and repeatedly experienced incidents of modest or slightly severe headaches in the past 4 weeks. A clinically significant shift to reduced migraine intervals in the KD intervention group was documented [99]. In addition, MAD was applied to adolescent individuals with prolonged daily headaches [100]. This survey was dismissed too early because of the lower adherence of participants to the diet. However, three individuals documented a lower headache intensity and a better quality of life, even if they even needed medication to cope with their disorder [100].

#### 3.2.9. Psychiatric Pathological Conditions

A cross-sectional analysis of clinical care was conducted on 31 individuals with serious, constant psychiatric disease (major depression, bipolar disease, and schizoaffective disease) whose symptomatology was inadequately controlled in spite of thorough psychiatric management [101]. The participants were assigned to a psychiatric hospital and followed a KD limited to a daily maximum of 20 g of carbohydrates as a complement to typical inpatient care [101]. The period of KD administration varied between 6 and 248 days. KD was linked to considerable improvements in depressive and psychotic symptomatology. Effect sizes were high concerning all mental health outcome rates in all participants’ groups and were even higher amongst those primarily diagnosed with major depression [101]. In addition, a case study was performed on a 65 year old woman who had simultaneously a 26 year history of non-insulin dependent diabetes and major depression disorder [102]. A 12 week KD personalized intervention attended to functionally inverse 26 years of diabetes mellitus, ameliorated two and a half decades of persistent depression disease, and empowered/equipped the patient with a new experience of optimism and positive completion [102].

In a case study, two female individuals with type II bipolar disease were capable of retaining ketosis for long durations (2 and 3 years, respectively) [103]. Both obtained mood stabilization that was better than that attained with drug therapy and showed a considerable subjective improvement that was clearly associated with ketosis, while they accepted the diet fairly [103]. These cases demonstrated that KD may be a viable approach for mood stabilization in type II bipolar disorder. It was also supported by the assumption that acidic plasma could enhance mood stabilization, possibly by decreasing intracellular Na and Ca [103]. Moreover, an analytical observational survey applied networked bipolar disease forums to obtain records from 141 individuals [104]. Eighty-five percent of the study individuals’ self-reported records showed helpful impacts on emotional state when adapting a KD [104]. Reports of emotional state maintenance or alleviation of symptoms were considerably greater for patients following KD than for patients following other dietary patterns like ω-3 fatty acid-enriched or vegetarian diets. Moreover, the enrolled individuals documented a reduction in depressive symptoms, better clarity of thoughts and oral communication, a weight decrease, and enhanced total energy [104].

The first indicative clinical evidence for the possible efficiency of KD in psychotic diseases was obtained 55 years ago in a small, open-label, uncontrolled clinical survey of 10 women hospitalized with schizophrenia [105]. In this study, the KD was complemented by medical therapy for 4 weeks. This study showed a substantial reduction in disease symptomatology following two weeks on KD [105]. In another case study, a 70-year-old female individual suffering from constant schizophrenia since her adolescence was documented to improve her schizophrenic symptoms considerably after adapting a KD for body weight decrease [106]. Within 8 days of beginning KD, she had no delusions and enhanced energy. After one year, her body weight decreased by 5 kg, and she stayed free of hallucinations [106].

More thorough and long-term case surveys have been performed in diverse populations and have presented promising findings concerning symptoms’ management, safety, and feasibility [107]. Two individuals with established, therapy-tolerant schizoaffective disease adopted KD for body weight reduction [106]. Within two months of beginning KD, both patients showed improvement in psychosis symptomatology as determined by the Positive and Negative Symptom Scale. Both individuals either intentionally or accidentally interrupted KD, and their psychotic symptoms reverted immediately [106]. Two additional case studies [108] highlighted the prolonged efficiency of KD as a balanced therapy that may lead to a complete reduction of psychotic symptoms, at least in certain individuals. The first case concerned a 12 year follow-up of a 70 year old woman [106]. At the age of 82, this woman, who experienced therapy-tolerant schizophrenia for 53 years, stayed active and well on the KD. She remained off all psychotropic medicines for 11 years, such as antipsychotic drugs, and stayed free of psychosis symptomatology. The other case concerned a 39 year old woman who showed therapy-resistant psychosis symptomatology for 20 years [106]. She adapted the KD following the instructions of a physician and, after quite a few months, exhibited full remission of her psychosis symptomatology. She was also capable of interrupting antipsychotic drugs and staying free of psychosis symptomatology for 5 years on the KD. Moreover, her social function was considerably enhanced [106]. Mechanistically, KD can change the fraction of GABA:glutamate in favor of GABA by inhibiting catabolism and enhancing GABA biosynthesis as well as glutamate metabolism, which may contribute to balancing the disturbed GABA concentrations in the schizophrenic brain, resulting in a better disease outcome regarding symptomatology [109].

In another clinical survey, 50 participating children and adolescents from a tertiary epilepsy center were randomly enrolled to a KD intervention group or a control group [110]. Follow-up evaluations concerning cognition and behavior were achieved almost four months after admission to KD, combined with parental report questionnaires and personally provided psychology assessments for the children [110]. The intervention group indicated decreased levels of anxiety and emotional state-disrupted behavior and was graded as more active. Cognitive assessment analysis indicated an increase in motivation in the intervention group [110].

#### 3.2.10. Stress-Related Pathologies

Stress urinary incontinence (SUI) restricts women’s daily lives and affects their quality of life, mainly for those affected by obesity. In a report of five cases, KD was utilized for the treatment of older female individuals diagnosed with SUI and simultaneously affected by obesity, and it was shown that KD might efficiently decrease body weight, ameliorate the symptoms of urine leakage, and reduce menopause symptoms [111].

## 4. Conclusions

Basic in vitro and in vivo research has revealed multiple molecular mechanisms through which KD can exert neuroprotective effects, such as neuroinflammation inhibition, decreased ROS production, lowered amyloid plaque accumulation and microglia triggering, protection in dopaminergic neurons, tau hyper-phosphorylation suppression, stimulating mitochondrial biogenesis, enhancing gut microbial diversity, induction of autophagy, restoration of histone acetylation, and neuron repair promotion.

On the other hand, clinical evidence remains scarce. Most existing clinical surveys are modest, usually without including a control group, and merely evaluate the short-term effects of KD. Moreover, several clinical studies had large dropout rates and a considerable lack of compliance assessment, as well as an increased level of heterogeneity concerning their design and methodological approaches. The above heterogeneity concerns age and sex fractions or individuals’ cognition states, which all exert a substantial impact on the probability of subsequent cognition impairment. The short follow-up periods and the repetitive cognition evaluations are predisposed to be potential contributing factors for a reexamination impact, mainly in cognitively unimpaired or MCI older adults. Inversely, individuals with mild-to-moderate dementia could be strictly diminished as well to achieve gains from a dietary intervention. Another concern is that the majority of surveys evaluating the impacts of dietary intervention on dementia or cognitive ability are performed by dietary questionnaires completed by individuals who already might exhibit problems recalling what they consumed or who present memory difficulties [112]. Thus, further studies are required to delineate whether the influence of KD in patients with neurodegenerative diseases may depend on the etiology of the illness by comparing the effects of the diet on patients with AD and PD and those with MS.

Moreover, several side effects can appear during ketosis, which are ascribed to metabolic modifications that occurred a few days after the beginning of the diet. This phenomenon is usually stated as “keto flu” and terminates naturally after a few days. The most commonly mentioned complications involve mental diseases like disturbed focusing as well as muscle pain, emotions of fragility and energy deficiency, and bloating or constipation [113].

Substantial evidence strongly supports the efficiency of KD in the management and therapy of epileptic pathology; however, this state is not comparable with other mental disorders. All meta-analyses and systematic reviews regarding AD, PD, and MS have been carried out in the last few years, supporting the necessity for further evaluation. Up to date, large-scale, longstanding clinical studies including participants’ randomization and control groups and assessing the effects of KD in people with neurodegenerative and psychiatric disorders remain scarce. Combined methods could be more efficient in preventing and/or slowing down these disorders, restraining disease development, and probably moderating disease symptomatology. Moreover, the currently available investigations of KD effects in patients with HD and stress-related pathologies remain extremely scarce, highlighting the need for future research in these fields.

A central disadvantage of KD is the use of ketone bodies in directed organs, mainly in the nervous system. The kinetics of ketone bodies seem to be highly influenced by the formulation and dosage of diverse KD remedies. Moreover, KD is very limiting [114] in comparison with other “healthy” dietary models, and its initiation is frequently related to various gastrointestinal complications such as constipation, diarrheic episodes, nausea, pancreatitis, and hepatitis, as well as hypoglycemia, electrolyte disturbances like hypomagnesemia and hyponatremia, and metabolic dysregulation evidenced by hyperuricemia or transient hyperlipidemia [115]. According to Taylor et al. [116], KD is able to be nutritionally compact, covering the Recommended Daily/Dietary Allowances (RDAs) of older adults. On the other hand, KD compliance necessitates intense daily adjustments, and, for this purpose, prolonged adherence is difficult and highly demanding to sustain [117]. For all these purposes, the periods of most KD interventions did not rise above six months.

The impact of KD on cognitive function appears promising; however, there are certain doubts concerning the efficient use of this dietary model in individuals diagnosed with mental diseases. In addition, comorbidities are very frequent among frail older adults, who are also at high risk of malnutrition during such restrictive diets. Among the most important features of KD is the decrease in desire for food, which could be related to stomach and intestine complications [118]. The above anorexic effect may also decrease eating quantities and total food consumption in aging individuals adapted to a KD, with the following enhanced probability of malnourishment and worsening of neurodegenerative symptomatology [117].

One more critical issue is the diversity of KD interferences applied in different study designs and methodologies. Moreover, several ketone salts are commercially accessible, and their major drawback deals with the fact that unhealthy salt consumption is needed to reach therapeutic doses of BHBA [119]. Endogenous and exogenous ketosis have their own possible advantages and disadvantages. Endogenous ketosis needs a more thorough metabolic shift, presenting the advantage of stimulating a wide range of metabolic pathways. Additionally, endogenous ketosis does not allow the specific targeting of ketone amounts, while exogenous ketosis does. There is also substantial data that both KD and exogenous ketone supplementation could support therapeutic advantages against neurodegenerative and psychiatric diseases. However, it remains uncertain which method is more effective than the other. In addition, a significant limitation of many KD studies is that many of them do not report the proportion of their sample that achieves nutritional ketosis. In this context, it should be noted that BHBA is a low-cost and easily obtainable biomarker of KD compliance. Most diets do not concern such a biomarker, and future clinical studies need to include this biomarker in their design and methodology to monitor nutritional ketosis conditions.

Furthermore, the specific food components of KD need to be considered since specific kinds of fat sources are healthier compared to others. Several types of KD necessitate rigorous monitoring of carbohydrate consumption, which frequently falls under the obligation of the caregiver. Thus, forthcoming surveys could be more advantageous in an institutional situation where it may be accessible to manage and adopt a strict nutritional protocol. Exogenous supplementation could be adapted easier as a prolonged remedy as the dietary adjustments are not so extreme. Conclusively, multidomain strategies and policies could be more efficient in preventing and/or delaying neurodegenerative and psychiatric diseases, alleviating disease progression, and improving quality of life.

## Data Availability

Not applicable.
